# The role of long-term power-law memory in controlling large-scale dynamical networks

**DOI:** 10.1038/s41598-023-46349-9

**Published:** 2023-11-09

**Authors:** Emily A. Reed, Guilherme Ramos, Paul Bogdan, Sérgio Pequito

**Affiliations:** 1 Ming Hsieh Electrical and Computer Engineering Department, University of Southern California, Los Angeles, USA; 2 Department of Computer Science and Engineering, Instituto Superior Técnico, University of Lisbon, Lisbon, Portugal; 3 Instituto de Telecomunicações, 1049-001 Lisbon, Portugal; 4 Division of Systems and Control, Department of Information Technology, Uppsala University, Uppsala, Sweden

**Keywords:** Electrical and electronic engineering, Complex networks

## Abstract

Controlling large-scale dynamical networks is crucial to understand and, ultimately, craft the evolution of complex behavior. While broadly speaking we understand how to control Markov dynamical networks, where the current state is only a function of its previous state, we lack a general understanding of how to control dynamical networks whose current state depends on states in the distant past (i.e. long-term memory). Therefore, we require a different way to analyze and control the more prevalent long-term memory dynamical networks. Herein, we propose a new approach to control dynamical networks exhibiting long-term power-law memory dependencies. Our newly proposed method enables us to find the minimum number of driven nodes (i.e. the state vertices in the network that are connected to one and only one input) and their placement to control a long-term power-law memory dynamical network given a specific time-horizon, which we define as the ‘time-to-control’. Remarkably, we provide evidence that long-term power-law memory dynamical networks require considerably fewer driven nodes to steer the network’s state to a desired goal for any given time-to-control as compared with Markov dynamical networks. Finally, our method can be used as a tool to determine the existence of long-term memory dynamics in networks.

## Introduction

Dynamical networks, including brain networks^1^, quantum networks^2^, financial networks^3^, gene networks^4^, protein networks^5^, cyber-physical system networks^6^ (e.g. power networks^2^, healthcare networks^7^), social networks^8^, and physiological networks^9^, exhibit not only an intricate set of higher-order interactions but also exhibit distinct long-term memory dynamics where both the recent and more distant past states influence the state’s evolution. Regulating these long-term memory dynamical networks in a timely fashion becomes critical to avoid a full-blown catastrophe. Examples include treating epilepsy by arresting a seizure in a human brain, mitigating climate-related power surges in the power grid, anticipating an undesired shock in the financial market, defending against cyber-attacks in cyber-physical systems, and even thwarting the spread of misinformation in social networks.

To address the challenge of regulating long-term memory dynamical networks, we need a general mathematical method to assess and design controllable large-scale long-term memory dynamical networks within a specified time horizon (i.e. time-to-control). More specifically, we need to be able to determine the minimum number of nodes in a long-term memory dynamical network that must connected to one and only one input to achieve a controllable dynamical network within a specified time horizon. These controlled nodes are known as driven nodes. While initial efforts on analyzing the controllability of long-term memory dynamic networks exist^10, 11^, these methods suffer from three main shortcomings. First, they do not assess the trade-offs between the time-to-control and the required number of driven nodes. Second, they require the knowledge of the exact parametrization of the system, which makes assessing such trade-offs computationally intractable. Third, these methods are limited to a few hundred nodes, which prohibits the analysis of real-world large-scale dynamical networks. In contrast, to overcome all the aforementioned limitations, we propose to analyze and design controllable large-scale long-term memory dynamical networks by considering the structure of the system that manifests when the exact system parameters are not known, which is both robust and scalable.

**Figure 1:**
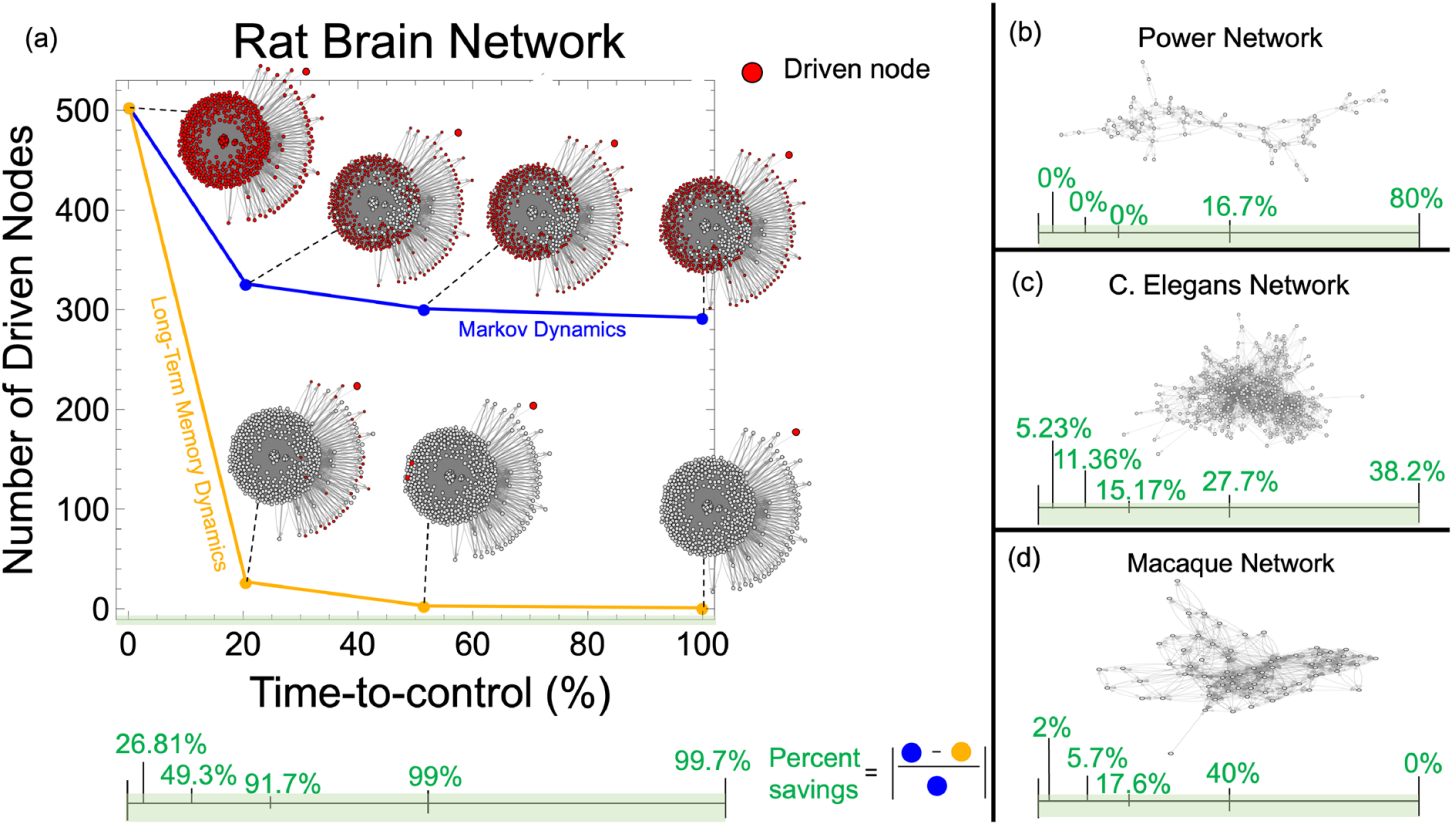
(**a**) The number of driven nodes is shown across the time-to-control for the rat brain network for both Markov and long-term memory dynamics. The rat brain network^12^ has 503 regions, which are captured by nodes in the network. At 0% of the time-to-control, all nodes in any dynamical network need to be driven nodes (shown in red). In just 20% of the time-to-control for the rat brain network, we see a drastic reduction in the required number of driven nodes for the long-term memory network as compared to the Markov dynamical network. As the time-to-control increases, the number of driven nodes decreases for both network dynamics; however, it is much more pronounced for the long-term memory dynamical network. The relationship between the percent savings (in green) and the time-to-control is shown at the bottom (highlighted in green). (**b**) shows the percent savings for the power network^13^ (60 nodes). (**c**) shows the percent savings for the C. Elegans network^14, 15^ (277 nodes). (**d**) shows the percent savings for the cortical brain structure of a macaque having 71 regions^16, 17^.

In particular, we present a strategy for determining the minimum number of driven nodes to control a long-term memory dynamical network within a specified amount of time, i.e. time-to-control.

Furthermore, our approach investigates the trade-off between the time horizon required to steer the network behavior to a desirable state and the required amount of resources necessary to correct the evolution of long-term dynamical networks. Subsequently, our approach provides answers to the following important questions: How does the nature of the dynamics (Markov versus long-term memory) affect the required number of driven nodes in networks having the same spatial topology? In the case of long-term power-law memory systems, how do the interactions between the nodes of a network, described as the spatial dynamics, affect the network’s ability to manipulate its evolution within a time frame to achieve a desired behavior? How does the size of a long-term memory dynamical network affect its ability to properly control itself on a specific time horizon? How does the inherent structure (i.e. topology) and its properties of a long-term power-law memory dynamical network affect its ability to quickly alter itself to operate correctly within a time horizon?

Figure 1 summarizes a few of the important outcomes of our proposed strategy. In short, given a network exhibiting either Markov or long-term memory dynamics, we compare the minimum number of driven nodes (in red) needed to steer the network to a desired goal within a given time horizon. Ultimately, our evidence suggests that long-term power-law memory dynamical networks require fewer driven nodes to steer the network to a desired behavior regardless of the given time-to-control.

## Methods

Fractional-order calculus and fractional-order dynamical networks provide efficient and compact mathematical tools for representing long-term memory with power-law memory dependencies^18–29^. These dependencies are captured by using a fractional-order derivative denoted by $\Delta ^{\alpha }$, which is the so-called Grünwald-Letnikov discretization of the fractional derivative, where $\mathbf {\alpha }= (\alpha _1,\alpha _2,\ldots ,\alpha _N)^{\textbf{T}}$ is the vector of fractional-order exponents. We represent a complex dynamical network exhibiting long-term memory as a fractional-order dynamical network. A fractional-order dynamical network is given as follows:

1 $$\begin{aligned} \Delta ^{\alpha }\textbf{x}[k+1] = A\textbf{x}[k]+B\textbf{u}[k], \end{aligned}$$

where time is discrete, so $k\in \{1,2,\ldots \}$, $\textbf{x}[k] = (x_1[k],x_2[k],\ldots ,x_N[k])^{\textbf{T}}\in \mathbb {R}^{N}$ is a state vector that assigns a single element to each node in the network, $\textbf{u}[k] = (u_1[k],u_2[k],\ldots ,u_N[k])^{\textbf{T}}\in \mathbb {R}^{N}$ is an input vector such that each element may influence a single state in the network, and *N* is the size of the network (i.e. the total number of nodes in the network).

The $N\times N$ matrix *A* describes the spatial relationship between the nodes in a network (i.e. the network topology). It is important to note that a network with nodal dynamics would mean that *A* has non-zero diagonal entries. The $N\times N$ matrix *B* describes the relationship between the external input and the nodes of the network, where we implicitly assume that one input element actuates only one node. This assumption limits the structure of the *B* matrix to be diagonal and potentially have non-zero diagonal entries.

**Figure 2:**
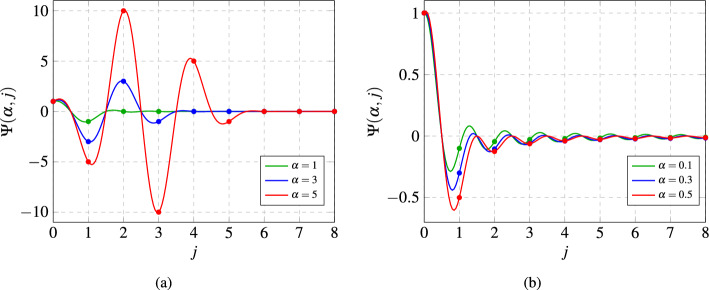
These figures show the behavior of the function $\Psi (\alpha ,j)$ for different values of $\alpha$. (**a**) shows the function of $\Psi (\alpha ,j)$ for integer values of $\alpha$. (**b**) shows the function of $\Psi (\alpha ,j)$ for non-integer values of $\alpha$. The non-zero values of $\Psi (\alpha ,j)$ for non-integer values of $\alpha$ enable the fractional-order system to capture long-term memory.

The vector of fractional-order exponents $\mathbf {\alpha }$ can capture the long-term memory of each state element assigned to each node in the network. This concept is evidenced by the following relationship for a single state at node *i*:

2 $$\begin{aligned} \Delta ^{\alpha _{i}}x_{i}[k] = \sum \limits _{j=0}^{k}\Psi (\alpha _{i},j)x_{i}[k-j], \end{aligned}$$

where $\Psi (\alpha _{i},j)$ encodes the weight or importance on each past state, and $\Psi (\alpha _{i},j) = \frac{\Gamma (j-\alpha _{i})}{\Gamma (-\alpha _{i})\Gamma (j+1)}$, with $\Gamma (\cdot )$ denoting the Gamma function^30^. We plot the behavior of the $\Psi (\alpha _i,j)$ function in Fig. 2.

We notice from (2) and Fig. 2b that as time increases (i.e. as *j* increases), the weights described by the function $\Psi (\alpha _i, j)$ will always be non-zero and decay according to a power-law as long as $\alpha _i$ is indeed a fractional value^18, 31^. Hence, each previous state $x_i[k-j]$ will be weighted with a non-zero power-law dependent value thereby forcing the dynamical network to be non-Markovian and possess what is referred to as long-term power-law dependent memory. In contrast, if $\alpha _i$ is instead an integer value, then the weights of $\Psi (\alpha _i, j)$ decrease to zero as *j* increases as seen in Fig. 2a, so the dynamical network will not possess long-term memory.

By combining (1) and (2), we obtain an equivalent formalism of the fractional-order dynamical network considered in this paper, which is given as follows^18, 31^:

3 $$\begin{aligned} x[k+1] = \sum \limits _{j=0}^{k}A_{j}x[k-j]+Bu[k], \end{aligned}$$

where $A_{0} = A-D(\alpha ,1)$ and $A_{j} = -D(\alpha , j+1)$, for $j \ge 1$ and

4 $$\begin{aligned} D(\alpha ,j) = \begin{bmatrix} \psi (\alpha _{1},j) &{} 0 &{} \dots &{} 0 \\ 0 &{} \psi (\alpha _{2},j) &{} \dots &{} 0 \\ 0 &{} \dots &{} \ddots &{} 0 \\ 0 &{} 0 &{} \dots &{} \psi (\alpha _{n},j) \end{bmatrix}. \end{aligned}$$

A fractional-order dynamical network described by system matrices $(\alpha , A, B,T)$ is *controllable* in *T* time steps, if there exists a sequence of inputs such that any initial state of the network can be steered to any desired state in a finite number of time steps *T*, where the time-to-control $T\in \{1,2,\ldots , N\}$ is the minimum time horizon necessary for the network to achieve controllability. If we want the network to achieve controllability in one time step, then all of the nodes must be driven nodes. On the other hand, if the time horizon equals the size of the network, then we obtain the minimum number of nodes needed to attain controllability.

The two most common strategies to assess controllability of complex networks are as follows: (i) quantitative and (ii) qualitative. Quantitative methods focus on the exact knowledge of the system parameters and seek to either compute the rank of the controllability matrix or the energy to control the network given by the Controllability Grammian^32^. The qualitative approach seeks to assess the possibility for controllability by assuming that the parameters are not known but the structure of the network is known (or, equivalently, which entries in the state space representation are nonzero). Such an approach is of practical use as often the network structure is the only information available. Qualitative methods often rely on structural systems theory^33^, as we also do in the current paper.

A structural matrix is defined as $\bar{M}=\{M\in \mathbb {R}^{m_1\times m_2} \ :\ \bar{M}_{i,j}=0 \text { if } M_{i,j}=0\}$, and $\bar{M}\in \{0,\star \}^{m_1\times m_2}$ is a structural matrix with fixed zeros and $\star$ represents an arbitrary scalar parameter. Subsequently, a fractional-order dynamical network with structural matrices $(\bar{\alpha }, \bar{A}, \bar{B}, T)$ is said to be *structurally controllable* in *T* time steps, if there exists a set $(\alpha , A, B, T)$ with the same structure as $(\bar{\alpha }, \bar{A}, \bar{B}, T)$ that is controllable.

Subsequently, any time-to-control $T\in \{1,2,\ldots , N\}$ is such that there exists $\bar{B}$ with

5 $$\begin{aligned} \text {g-rank}(\mathscr {C}(\bar{A}, \bar{B}; T)) = N, \end{aligned}$$

where g-rank is the generic rank of an $n\times m$ structural matrix $\bar{M}$ given as

6 $$\begin{aligned} \text {g-rank} = \max _{M\in [\bar{M}]}\text {rank}(M), \end{aligned}$$

where $[\bar{M}]=\{M\in \mathbb {R}^{n\times m}: \bar{M}_{i,j}=0 \text { if } {M}_{i,j}=0\}$, and $\mathscr {C}$ is the structural controllability matrix given by

7 $$\begin{aligned} \mathscr {C}(\bar{A},\bar{B}; T) = [\bar{B} \qquad \bar{A}\bar{B} \qquad \cdots \qquad \bar{A}^{T-1}\bar{B}]. \end{aligned}$$

Hereafter, we seek to determine the minimum number of driven nodes that ensure that $(\bar{\alpha }, \bar{A},\bar{B},T)$ is structurally controllable in *T* time steps. Specifically, we aim to determine $\bar{B}$ with the minimum number of non-zero diagonal entries such that a given fractional-order dynamical network, represented as $(\bar{\alpha }, \bar{A}, \bar{B}, T)$, is structurally controllable in *T* time steps. We achieve this through the following result, which relates the number of driven nodes required to control fractional-order dynamical networks to the number of driven nodes required to control Markov dynamical networks – see Supplementary Material for details.

### Theorem 1

The minimum number of driven nodes required to structurally control a fractional dynamical network within a specified time-to-control is generically equal to that of a Markov dynamical network where all nodes possess self-loops.

The nodal dynamics that emerge from analyzing the fractional-order dynamics are a result of Theorems 2 and 3 in the Supplementary Material, which relate the structural equivalence of the fractional-order system to a linear time-invariant system having nodal dynamics. These results are key in finding the conditions for structural controllability of fractional-order dynamical networks and hence fundamental in providing a solution for the minimum number of driven nodes for long-term memory dynamical networks.

There are two significant consequences from Theorem 1. First, if a network has self-loops on every node, then the dynamical networks with and without long-term power-law memory require the same number of driven nodes for the same time-to-control horizon, and subsequently, in some sense, they are equally difficult to control. Second, networks with long-term power-law memory dynamics are always, at most, as difficult to control as Markov dynamical networks that have the same network topology. Nonetheless, it is surprising that the minimum number of driven nodes for a network possessing long-term power-law memory dynamics can be significantly lower than the same topological network possessing Markov dynamics without self-loops. Hence, it may be easier to control long-term memory networks than the corresponding Markov networks that do not contain self-loops.

In general, the time-to-control can be greater than the size of the network, but because we are interested in understanding structural controllability properties, our results establish that generically speaking one can design a controllable fractional dynamical network by designing a controllable linear time-invariant dynamical network with nodal dynamics. Hence, from the results for linear time-invariant dynamical networks, we know that if the system is controllable, then it must be controllable in at most *N* steps (the size of the network) by Cayley–Hamilton’s theorem (Theorem 12.1^34^).

Given a network, we define the difference in the number of driven nodes as follows:

$$\begin{aligned} n_T=n_{\text {Markov}}-n_{\text {long-term memory}}, \end{aligned}$$

where $n_{\text {Markov}}$ is the number of driven nodes needed to control the network when it possesses Markov dynamics and $n_{\text {long-term memory}}$ is the number of driven nodes needed to control the network when it possesses long-term memory dynamics.

We define the percent difference $(\% D)$ as follows:

$$\begin{aligned} \% D = \frac{n_T}{N}\times 100, \end{aligned}$$

where $n_T$ is the difference in the number of driven nodes and *N* is the size of the network. We also define the percentage of the time-to-control ($\%$ time-to-control) as follows.

$$\begin{aligned} \% \text { time-to-control} = \frac{\text {T}}{N}\times 100, \end{aligned}$$

where $T\in \{1,2,\ldots ,N\}$ is defined as the minimum time horizon needed to control a network and *N* is the size of the network. Finally, we define the percent savings as

$$\begin{aligned} \% \text { savings} = \frac{n_T}{n_{\text {Markov}}}\times 100, \end{aligned}$$

where $n_T$ is the difference in the number of driven nodes and $n_{\text {Markov}}$ is the number of driven nodes needed to control the network when it possesses Markov dynamics.

In what follows, we conduct a detailed study to further understand the variations in the number of driven nodes for different dynamics, time horizons, and network topologies. We examined real-world and synthetic networks, including Erdős–Rényi, Barabási–Albert, and Watts-Strogatz networks^35^. To generate the Erdős–Rényi network, we selected a network uniformly at random from the collection of all networks having a pre-defined number of nodes and edges. The Barabási–Albert network is a scale-free network with two parameters, the number of nodes and a parameter *k*, where a new vertex with *k* edges is added at each step in generating the network. Finally, the Watts-Strogatz network exhibits small-world properties, including high clustering and short average path lengths. The two parameters for generating Watts-Strogatz networks include the number of nodes and the rewiring probability *p*. The adjacency matrix associated with the network topology forms the matrix $\bar{A}$.

## Results

We performed experiments on both synthetic and real-world networks. In each experiment, we find the difference between the required number of driven nodes ($n_T$) for the network having Markov dynamics and for the same network having long-term memory dynamics in a given time-to-control. Since the available time-to-control varies with the size of a particular network, we consider the percentage of the time-to-control (% time-to-control).

**Figure 3:**
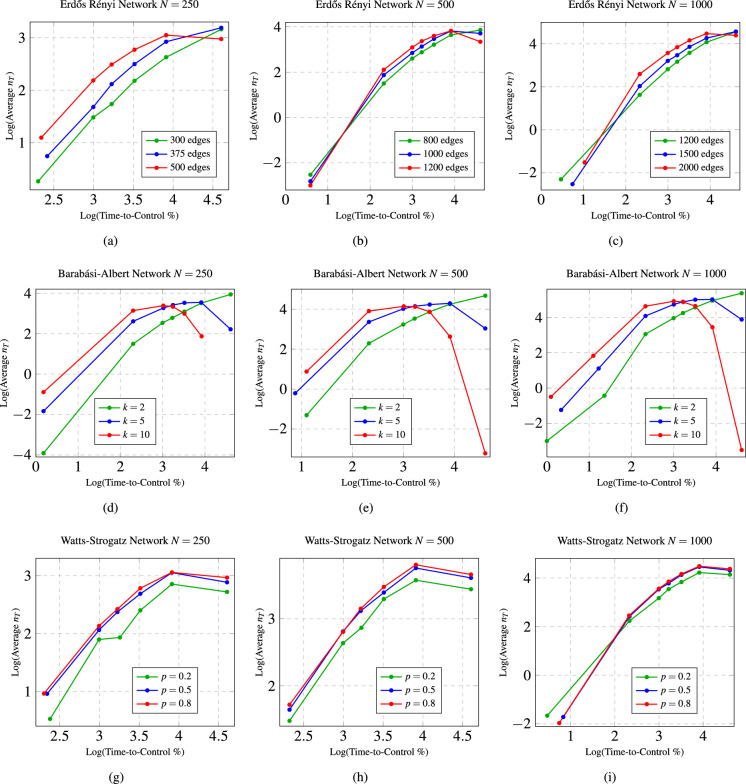
These figures show the relationship between the average difference (over 100 networks) in the required number of driven nodes across the time-to-control for different types of synthetic networks with different sizes and parameters. For network sizes 250, 500, and 1000, respectively, (**a**–**c**) show the log-log plot of the average difference in the required number of driven nodes ($n_T$) versus the time-to-control (%) for 100 realizations of Erdős–Rényi networks with various edge parameters. For network sizes 250, 500, and 1000, respectively, (**d**–**f**) show the log-log plot of the average difference in the required number of driven nodes ($n_T$) versus the time-to-control (%) for 100 realizations of Barabási–Albert networks with various *k* parameters. For network sizes 250, 500, and 1000, respectively, (**g**–**i**) show the log-log plot of the average difference in the required number of driven nodes ($n_T$) versus the time-to-control (%) for 100 realizations of Watts-Strogatz networks with various *p* parameters.

**Figure 4:**
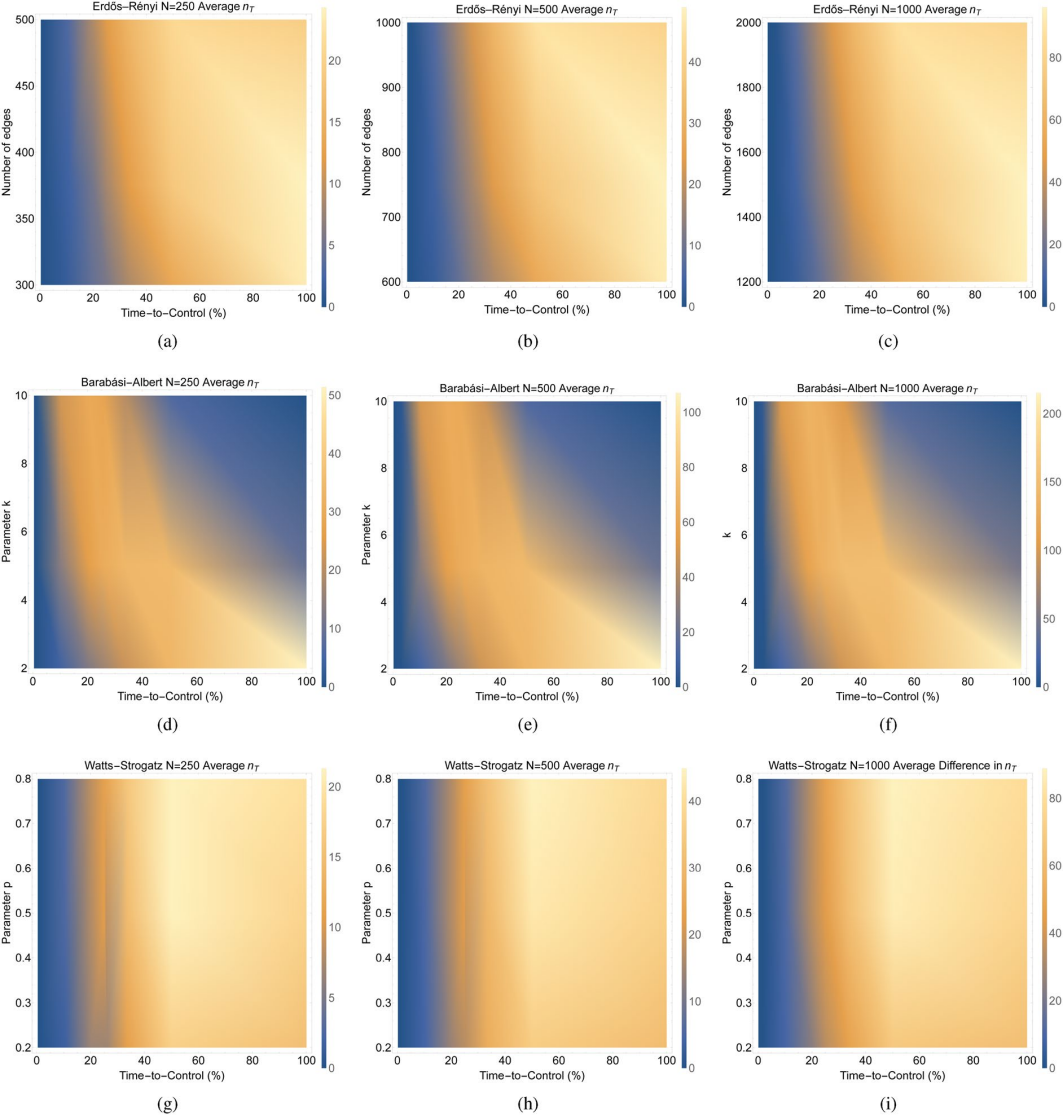
These figures plot the average difference (over 100 networks) in the required number of driven nodes ($n_T$) across the time-to-control ($\% \text { time-to-control}$) for different types of random networks having different sizes and parameter values. For network sizes 250, 500, and 1000, respectively, (**a**–**c**) show average difference in the required number of driven nodes ($n_T$) across the number of edges in the network versus the time-to-control (%) for 100 realizations of Erdős–Rényi networks. For network sizes 250, 500, and 1000, respectively, (**d**–**f**) show the average difference in the required number of driven nodes ($n_T$) across the *k* parameter versus the time-to-control (%) for 100 realizations of Barabási–Albert networks. For network sizes 250, 500, and 1000, respectively, (**g**–**i**) show the average difference in the required number of driven nodes ($n_T$) across the *p* parameter versus the time-to-control (%) for 100 realizations of Watts-Strogatz networks.

**Figure 5:**
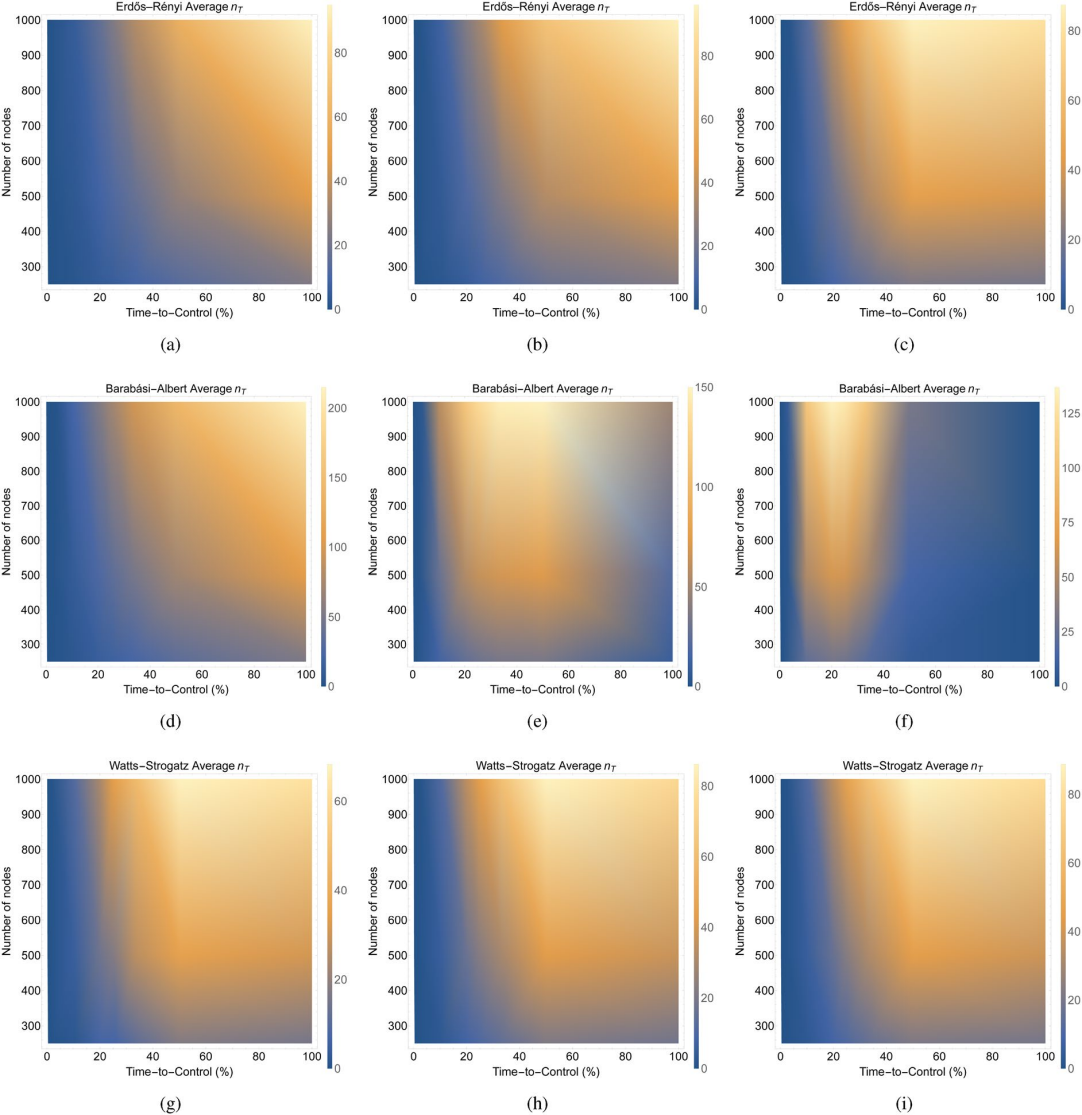
These figures plot the average difference (over 100 networks) of the required number of driven nodes across the time-to-control for different types of random networks having different sizes and parameter values. For the the edge count being 20%, 50%, and 100% higher than the number of nodes, respectively, (**a**–**c**) show the average difference in the required number of driven nodes ($n_T$) along the size of the network versus the time-to-control (%) for 100 realizations of Erdős–Rényi networks. For parameter value $k=2,5,10$, respectively, (**d**–**f**) show the average difference in the required number of driven nodes ($n_T$) along the size of the network versus the time-to-control (%) for 100 realizations of Barabási–Albert networks. For parameter value $p=0.2,0.5,0.8$, respectively, (**g**–**i**) show the average difference in the required number of driven nodes ($n_T$) along the size of the network versus the time-to-control (%) for 100 realizations of Watts-Strogatz networks.

**Figure 6:**
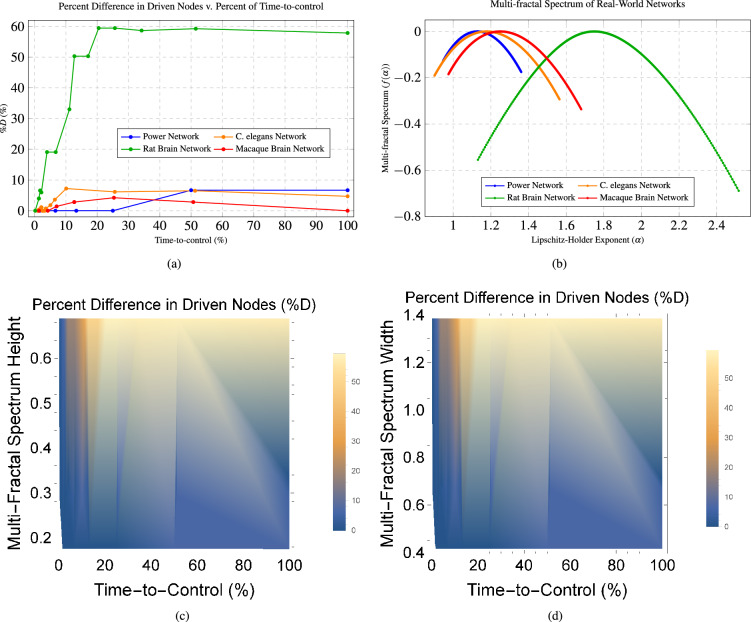
These figures show the relationship between the percent difference in the number of driven nodes across the time-to-control (%) and the multi-fractal spectrum of four real-world networks, including a power network (60 nodes), rat brain network^12^ (503 nodes), C. elegans network^14, 15^ (277 nodes), and a macaque brain network^16, 17^ (71 nodes). (**a**) shows the plot of the percent difference of the required number of driven nodes ($n_T$) versus the percent of time-to-control (%) for several real-world networks. (**b**) shows the plot of the multi-fractal spectrum of several real-world networks. (**c**) shows the spectrum of the difference in the required number of driven nodes ($n_T$) compared with the multi-fractal spectrum width and the time-to-control ((%) for the same four real-world networks. (**d**) shows the spectrum of the difference in the required number of driven nodes ($n_T$) compared with the multi-fractal spectrum height and the time-to-control (%) for the same four real-world networks.

### Long-term memory dynamical networks require fewer driven nodes than Markov counterparts

Our experimental results confirm our theoretical results that long-term power-law memory dynamical networks require equal or fewer driven nodes than Markov dynamical networks having the same structure. In all of the experiments, we find that the difference in the required number of driven nodes ($n_T$) is non-negative. In fact, the experiments for the Erdős–Rényi networks (see Fig. 3a–c) suggest that the average (over 100 random networks) difference in the number of driven nodes across the time-to-control partially resembles a power-law. For the Barabási–Albert networks (Fig. 3d–f), the average difference in the number of driven nodes initially scales by a power-law but then quickly drops very low nearly to zero difference as the time-to-control increases. Finally, the average difference in the number of driven nodes for the Watts-Strogatz networks (Fig. 3g–i) scales initially by a power-law and then levels out to a linear relationship towards the final time-to-control. The results in Fig. 3 show that, depending on the network topology, a Markov dynamical network requires up to a power-law times more driven nodes to achieve structural controllability than a long-term memory dynamical network having the same spatial structure.

For several real-world dynamical networks, we notice similar trade-offs between the difference in the number of driven nodes and the time-to-control see Fig. 6a. We find that the rat brain network^12^ gives approximately a 60% difference in the minimum number of driven nodes, which is achieved around 20% of the time-to-control (Fig. 6a). These results are significant considering that the rat brain network has 503 total nodes. The *Caenorhabditis elegans* (C. elegans) network^14, 15^, which has 277 nodes, has approximately a 12% difference in the number of driven nodes at around 20% of the time-to-control (Fig. 6a). However, the percent difference in the number of driven nodes decreases as the time-to-control increases in the C. elegans network. The power network^13^, with only 60 nodes, has approximately an 8% difference in the number of driven nodes at the final time-to-control (Fig. 6a). Lastly, the macaque brain network^16, 17^, containing 71 regions (and, subsequently 71 nodes), gives less dramatic results with only a peak of 5% difference in the number of driven nodes achieved at the 25% time-to-control (Fig. 6a). These real-world networks show the capability of saving up to 91% of resources as early as 20% of the total time-to-control when controlling large-scale networks exhibiting long-term memory dynamics.

### Network topologies, not size, determine the control trends

We generated 100 random networks having the same size and synthetic parameters and found the average difference in the number of driven nodes ($n_T$) versus the time-to-control (see Fig. 3). The results in Fig. 3 show that the same overall trend occurs for each of the three types of random networks (Erdős–Rényi, Barabási–Albert, and Watts-Strogatz) independent of the network size. These results suggest that the network topology significantly affects the controllability over other network attributes, such as the size of the network.

We examine the effect of varying the parameters on the average difference in the required number of driven nodes across the time-to-control for the three types of random networks. Across the sizes of the networks, we notice similar trends for the Erdős–Rényi and Watts-Strogatz networks and drastically different results for the Barabási–Albert networks (Fig. 4). The results in Fig. 4 suggest that varying the number of edges for the Erdős–Rényi networks and the *p* parameter for the Watts-Strogatz networks, in general, show similar behavior in their difference in the number of driven nodes across the time-to-control. However, for the Barabási–Albert network, varying the *k* parameter greatly impacts the difference in the number of driven nodes across the time-to-control. For example, on the one hand, a lower *k* parameter in the Barabási–Albert network gives a higher difference in the number of driven nodes at higher time-to-control values. On the other hand, a higher *k* parameter gives a lower difference in the number of driven nodes at higher time-to-control values (Fig. 4). For each of the different random networks, as the network sizes increase, the same pattern appears but the difference in the required number of driven nodes increases proportionally with the network size. These results provide further evidence that the topology of the network has a significant influence on the controllability of the network.

We aim to analyze the impact of varying network size on the average difference in the required number of driven nodes over the time-to-control for distinct types of random networks. In Fig. 5, we show the average difference (computed across 100 networks) in terms of the required number of driven nodes. The focus is on different types of random networks, each having varying sizes and parameter values.

Specifically, for networks with edge counts 20%, 50%, and 100% higher than the number of nodes, we plot the average difference in the required number of driven nodes ($n_T$) as a function of both the network size and the time-to-control (%) in Fig. 5a–c from 100 instances of Erdős–Rényi networks. Similarly, with parameter values $k=2$, $k=5$, and $k=10$, we illustrate the average difference in the required number of driven nodes ($n_T$) against network size and time-to-control (%) in Fig. 5d–f based on 100 realizations of Barabási–Albert networks. Furthermore, we show the average difference in the required number of driven nodes ($n_T$) against the network size and time-to-control (%) for 100 instances of Watts-Strogatz networks, using parameter values $p=0.2$, $p=0.5$, and $p=0.8$, in Fig. 5g–i.

Observing the Erdős–Rényi and Watts-Strogatz networks,﻿ in Fig. 5, we identify a pattern where the difference in the required number of driven nodes increases with both network size and time-to-control. These trends hold true across different parameter values. In contrast, the variability in the number of driven nodes for Barabási–Albert networks is heavily influenced by the parameter *k*. These results support the claim that the network topology plays a relevant role in determining the controllability of the network, whereas the network size does not seem to play a relevant role.

### Multi-fractal spectrum dictates savings when controlling long-term memory dynamical networks

From the results in Cowan et al.^36^, the degree distribution of a network does not determine the required number of driven nodes to ensure controllability. Nonetheless, we analyze the relationship between the average degree of a network, the difference in the required number of driven nodes, and the time-to-control in Fig. 1 in the Supplementary Material for differently sized random networks. These results suggest that more than the average degree, the topology of the network plays a larger role in determining the difference in the required number of driven nodes.

Knowing these results, we focus on studying the relationship between the multi-fractal spectrum and the difference in the required number of driven nodes since the multi-fractal spectrum is a measure of the heterogeneity in the structure of the network^37, 38^. We investigate the relationship between the multi-fractal spectrum and the difference in the required number of driven nodes in several real-world networks, namely the rat brain^12^, C. elegans^14, 15^, macaque brain^16, 17^, and power networks^13^. We pay particular attention to the width of the spectrum $w=\alpha _{\max }-\alpha _{\min }$, where $\alpha _{\max }$ is the maximum Lipschitz–Holder exponent and $\alpha _{\min }$ is the minimum Lipschitz–Holder exponent, since the width reflects the degree of structural heterogeneity^39^. Thus, the wider the spectrum, the more heterogeneous the network structure will be. The results indicate that, in general, the wider and higher the multi-fractal spectrum, the larger the percent difference in the number of driven nodes – see Fig. 6a and b. From our results in Fig. 6a and b, it seems that the difference in the number of driven nodes is proportional to the structural heterogeneity of a network, meaning that, the wider the multi-fractal spectrum, the larger the difference in the number of driven nodes. This conclusion is also supported by Fig. 6d, which plots the relationship between the multi-fractal spectrum width, time-to-control, and percent difference in the number of driven nodes for the four real-world networks.

Figure 6c plots the relationship between the multi-fractal spectrum height, time-to-control, and percent difference in the number of driven nodes for the four real-world networks. The height of the multi-fractal spectrum indicates the frequency of a particular network structure^39^. Hence, the higher the multi-fractal spectrum, then the more structures that appear in the network. These figures, Fig. 6c and d, support the notion that, in general, the greater the width and height of the multi-fractal spectrum, then the greater the difference in the number of driven nodes for nearly all time-to-control horizons. Hence, the greater the structural heterogeneity of the network, then the greater the savings in the amount of driven nodes when controlling long-term memory dynamical networks. Therefore, the width of the multi-fractal spectrum is an indicator of the total savings of driven nodes that can be achieved when controlling long-term memory dynamical networks, and the multi-fractal spectrum is more indicative of the difference in the required number of driven as compared with the average degree.

## Discussion

### Fewer required driven nodes, dictated by network topology and multi-fractal spectrum

Following from the results of Theorem 1, we proved that a long-term power-law memory requires less than or equal to the number of driven nodes as the same network possessing Markov dynamics requires (see Theorem 6 in the Supplementary Material). In our experimental results, we showed that long-term power-law memory dynamical networks can provide a significant advantage in terms of the resources for controlling networks. In particular, the results in Fig. 3 showed that, depending on the network topology, a Markov dynamical network requires up to a power-law times more driven nodes to achieve structural controllability than a long-term power-law memory dynamical network having the same spatial structure.

Long-term power-law memory dynamical networks can use 91% fewer driven nodes at 20% of the time-to-control compared to Markov dynamical networks. More specifically, the rat brain network showed a 60% difference in the minimum number of driven nodes at approximately 20% of the time-to-control (Fig. 6a). Hence, we provided evidence that long-term power-law memory dynamical networks may be easier to control than Markov dynamical networks.

Our experimental results showed that the network topology, not the size of the network, determined the overall trends of the difference in the minimum resources needed for control over the time-to-control (see Figs. 3, 4 and 5). The results in Fig. 3 showed that the same overall trend occurs for each of the three types of random networks (Erdős–Rényi, Barabási–Albert, and Watts-Strogatz) independent of the network size. The results in Fig. 4 suggest that the same pattern appears but the difference in the required number of driven nodes increases proportionally with the network size for each of the different random networks. Finally, the results in Fig. 5 showed that the difference in the minimum number of driven nodes increased as the time-to-control increases for both the Erdős–Rényi and Watts-Strogatz networks, whereas the difference in the minimum number of driven nodes for the Barabási–Albert network is heavily dependent on the parameter *k*.

It is difficult to determine which nodes are better to control because it depends on the specific connections within the network. To illustrate this fact, we notice that the algorithm that we develop and analyze in this paper depends on first partitioning the network and then on identifying the *source* strongly components within these partitions. Therefore, our algorithm requires that one node per *source* SCC in every partition is selected to be controlled. A graph can be partitioned in multiple different ways as outlined in Pequito et al.^40^ and, identifying these partitions and their source strongly connected components are not not correlated with other network properties such as network communities and metrics^41^. Hence, it becomes difficult to describe which nodes are best to be controlled.

Remarkably, in contrast with the work by Liu et al.^42^, the *difference* in the minimum number of driven nodes to achieve controllability for an arbitrary time-to-control does not correlate with the degree distribution of the network. We provide evidence to support this claim in Fig. 2 in the Supplementary Material. Here, we generated 100 networks having a similar degree distribution to the rat brain network and computed the difference in the number of driven nodes over the time-to-control. However, the difference in the number of driven nodes for 100 generated networks having a similar degree distribution to the rat brain network varies drastically from the difference in the number of driven nodes for the original rat brain network. Hence, having a similar degree distribution does not necessarily mean that the difference in the number of driven nodes will be similar across the time-to-control. A related conclusion demonstrated that the degree distribution of a linear time-invariant dynamical network does not dictate the nodes that must be controlled^36^.

Finally, we find that the height and width of the multi-fractal spectrum serve as an indication of the total savings in the minimum number of driven nodes for a given network, when considering long-term memory dynamics over Markov dynamics (see Fig. 6). In particular, Fig. 6c and d, support the notion that, in general, the greater the width and height of the multi-fractal spectrum, then the greater the difference in the minimum number of driven nodes for nearly all time-to-control horizons.

### A scalable and robust method to determine the trade-offs between the minimum number of driven nodes and time-to-control for controlling large-scale long-term power-law memory dynamical networks

The work by Pequito et al.^43^ characterized the minimum number of driven nodes for controlling Markov networks and used these results to provide algorithms to design controllable Markov networks. Liu et al. showed the relationship between the required number of driven nodes in Markov networks and the average degree distribution for different network topologies^42^. The work in^36^ showed the importance of nodal dynamics in determining the minimum number of inputs (i.e. an input can be connected to multiple nodes) to achieve structural controllability in the case of Markov networks.

Recent work by Lin et al. claims to control Markov networks with only two time-varying control inputs with the assumption that these sources can be connected to any node at any time; however, this is not a realistic solution in the case of many physical systems, such as the power grid or the brain^44^. Most notably though, none of these previously mentioned works take into account the long-term memory dynamics exhibited in many real-world complex dynamical networks. Furthermore, only the work by Pequito et al.^40^ has analyzed the trade-offs between the time-to-control of a dynamical network and the required number of driven nodes, but this study is limited to only considering Markov dynamical networks. Here, we have examined the effect that long-term memory dynamics plays on the ability to control the network while considering the time-to-control.

There are only two works^10, 11^ that have examined the control of networks exhibiting long-term memory dynamics, but they have several shortcomings, including scalability and lack of robustness. For example, the work in by Kyriakis et al.^10^, which uses energy-based methods to control long-term memory dynamics modeled as a linear time-invariant fractional-order system in discrete-time, is not tractable as it has computational-time complexity of $\mathscr {O}(n^{7.748})$. Similarly, the work by Cao et al.^11^, which uses a greedy algorithm that maximizes the rank of the controllability matrix, relies on the computation of the matrix rank, so it quickly becomes intractable as a network grows in size and has computational-time complexity of $\mathscr {O}(n^5)$. Furthermore, both of these works require that the precise dynamics are known^10, 11^. Simply speaking, these works do not account for the inherent uncertainty in the parametric model for long-term memory dynamics.

Hence, we present the first scalable and robust method to determine the trade-offs between the minimum number of driven nodes and time-to-control for controlling large-scale long-term power-law memory dynamical networks with a computational-time complexity of $\mathscr {O}(n^2\log (n))$ – See Theorem 11 in the Supplementary Material. Our novel method can assess the trade-offs in controlling a large-scale long-term memory dynamical network with unknown parameters in a given number of time steps, which leads us to provide answers to fundamental questions regarding the relationships between the number of driven nodes, the time-to-control, the network topology, and the size of the network.

### Determining the existence of long-term memory in dynamical networks

Our approach, which assesses the trade-offs between the minimum number of driven nodes in a given time-to-control for controlling long-term power-law dynamical networks, can determine the existence of long-term memory in dynamical networks. We can achieve this by considering whether a network can be controlled with the minimum number of driven nodes given by the proposed method.

For example, suppose that we want to control a dynamical network in a finite amount of time, where we assume that we know the structure of the network including its size (i.e. number of nodes) and the relationship between nodes in the network (i.e. the edges and their placement in the network), but we do not know the dynamics of the system. In this case, our approach can be used to determine whether the dynamical network possesses long-term memory by first trying to control the network with the minimum number of driven nodes for long-term memory dynamics in a specified time frame, which can be computed using our proposed approach. If the dynamical network can indeed be steered to a desired behavior with the minimum number of driven nodes in a given time-to-control as computed by our proposed method, then the dynamical network indeed possesses long-term power-law memory dynamics. On the other hand, if the network cannot be controlled with the minimum number of driven nodes in a specified time frame as computed using our proposed method, then the network must not possess long-term power-law memory dynamics as it requires more driven nodes to control the network.

This is a powerful result that provides a significant advantage over current state-of-the-art methods to determine the existence of long-term memory dynamics in large-scale networks, which rely on first finding the exact parameterization of the known dynamics of the system from data^6, 45–47^. In particular, these approaches estimate the two parameters of the long-term power-law dynamical networks, namely the fractional-order exponents ($\mathbf {\alpha }$) and the spatial matrix (*A*). By investigating whether the estimated values of the fractional-order exponents are indeed fractional, this inherently determines the existence of long-term memory in the dynamics (see Fig. 2).

There are several works that have proposed methods to estimate the parameters of long-term power-law dynamical networks. The work by Xue et al.^6^ proposes an approximate approach to estimate the fractional-order exponents, then based on this result, the methods finds the spatial matrix using a least-squares approach. The work by Flandrin et al.^48^ leverages wavelets to find the fractional-order exponents. Using this wavelet approach, the work by Gupta et al.^45^ proposes an expectation-maximization approach to estimate the the spatial matrix and the unknown inputs of a fractional-order system. In a similar vein, the work in by Gupta et al. ^46^ proposes a method to estimate from data the parameters of a fractional-order system having partially unknown states. Finally, the work by Chatterjee et al. ^47^ considers a finite-sized augmented fractional-order system and proposes an iterative algorithm to find the fractional-order exponents and a least-squares approach to find the spatial matrix.

While these works rely on computing the parameters of long-term power-law dynamical networks from data, the advantage of our proposed approach is that it can determine the existence of long-term memory dynamics in large-scale networks without knowing the exact dynamics of the system.

### Supplementary Information

Supplementary Information.

## Data Availability

The real-world networks, that is the rat brain^12^, C. elegans^14, 15^, macaque brain^16, 17^, and power networks^13^, are publicly available. The code is available upon request by contacting the corresponding author.
