# Supplementary material for: The role of long-term power-law memory in controlling large-scale dynamical networks
Source: Sci Rep. 2023 Nov 9;13:19502. doi: 10.1038/s41598-023-46349-9 (PMC10636034; doi:10.1038/s41598-023-46349-9)
Supplement: Supplementary file 1 — Supplementary Information. [file 41598_2023_46349_MOESM1_ESM.pdf]

Supplementary Material of The Role of Long-Term Power-Law Memory in Controlling Large-Scale Dynamical Networks

Emily A. Reed, Guilherme Ramos, Paul Bogdan, and Sérgio Pequito

Contents

1 Methods **1**

2 Extra Experiments **7**

1 Methods

Problem Statement

We consider a fractional-order dynamical network driven by a control input and additive noise. It is described as follows:

$$\Delta^{\alpha}\mathbf{x}[k+1] = A\mathbf{x}[k] + B\mathbf{u}[k], \tag{1}$$

where $k \in \mathbb{N}$ is the time step, n is the number of nodes in the network, $x[k] \in \mathbb{R}^n$ denotes the state, $A \in \mathbb{R}^{n\times n}$ is the coupling matrix that describes the spatial relationship between different states, $\mathbf{u}[k] \in \mathbb{R}^n$ is the input vector, $B \in \mathbb{R}^{n\times n}$ is the coupling matrix that describes the spatial relationship between the inputs and the states, $\alpha \in \mathbb{R}^n$ are the fractional-order exponents encoding the memory associated with the different state variables, and Δ^{α} is the Grünwald-Letnikov discretization of the fractional derivative (Chpt.2,[1]). Fractional-order dynamical networks possess long-term memory. For each i-th state ($1 \leq i \leq n$), the fractional-order operator acting on x_i leads to the following expression:

$$\Delta^{\alpha_i} x_i[k] = \sum_{j=0}^{k} \psi(\alpha_i, j) x_i[k-j], \tag{2}$$

where $\psi(\alpha_i, j) = \frac{\Gamma(j-\alpha_i)}{\Gamma(-\alpha_i)\Gamma(j+1)}$, with $\Gamma(\cdot)$ denoting the Gamma function [2].

We aim to determine the minimum number of state nodes and their placement that need to be driven to ensure the structural controllability of the fractional-order dynamical network. A fractional-order dynamical network is said to be *controllable* if there exists a sequence of inputs such that any initial state of the system can be steered to any desired state in a finite number of time steps. Therefore, assuming that the system is being actuated during T time steps, we can describe the system (1) by the matrix tuple (α, A, B, T).

Controllability associated with the system described in (1) can be characterized as follows.

Definition 1. *(Controllability in T time steps) The fractional-order dynamical network described by (α, A, B, T) is said to be controllable in T time steps if and only if there exists a sequence of inputs $\mathbf{u}[k]$ $(0 \leq k \leq T-1)$ such that any initial state $\mathbf{x}[0] \in \mathbb{R}^n$ can be steered to any desired state ($\mathbf{x}_{desired}[T] \in \mathbb{R}^n$) in T time steps.* ∘

Next we provide the following result on the controllability of the linear discrete-time fractional-order dynamical network.

Theorem 1. *(Controllability of fractional-order dynamical network (Theorem 4, [3])) The linear discrete-time fractional-order dynamical network is controllable if and only if there exists a finite time K such that* $rank(W_c(0,K)) = N$*, the dimension of the state, where* $W_c(0,K) = G_K^{-1}\sum_{j=0}^{K-1} G_j BB^\intercal G_j^\intercal G_K^{-\intercal}$ *and*

$$G_k = \begin{cases} I_n, & k = 0 \\ \sum_{j=0}^{k-1} A_j G_{k-1-j}, & k \geq 1, \end{cases} \tag{3}$$

where I_n is the identity matrix of size n. ◦

Furthermore, an input sequence $\left[\mathbf{u}^\intercal[K-1], \mathbf{u}^\intercal[K-2], \ldots \mathbf{u}^\intercal[0]\right]^\intercal$ that transfers $\mathbf{x}[0] \neq 0$ to $\mathbf{x}[K] = 0$ is given by

$$\begin{bmatrix} \mathbf{u}[K-1] \\ \mathbf{u}[K-2] \\ \vdots \\ \mathbf{u}[0] \end{bmatrix} = -[G_0 B G_1 B \ldots G_{K-1}B]^\intercal G_K^{-\intercal} W_c^{-1}(0,K)\mathbf{x}[0]. \tag{4}$$

Due to the uncertainty in the system's parameters, we adopt a structural systems approach that relies solely on the system's parameters. Consider the class of possible tuples with a predefined structure $([\bar{\alpha}], [\bar{A}], [\bar{B}], T)$, with $[\bar{\alpha}] = \{\alpha \in \mathbb{R}^n\}$, where a structural matrix is defined as $[\bar{M}] = \{M \in \mathbb{R}^{m_1 \times m_2} \ : \ \bar{M}_{i,j} = 0 \text{ if } M_{i,j} = 0\}$, and $\bar{M} \in \{0, \star \in \mathbb{R}\}^{m_1 \times m_2}$ is a structural matrix with fixed zeros and arbitrary scalar parameters. Specifically, in the context of this paper, we seek to assess the *structural controllability* defined as follows:

Definition 2. *(Structural Controllability): The fractional-order dynamical network with structural pattern $(\bar{\alpha}, \bar{A}, \bar{B}, T)$ is said to be structurally controllable in T time steps if and only if there exists a tuple $(\alpha', A', B', T) \in ([\bar{\alpha}], [\bar{A}], [\bar{B}], T)$ that is controllable in T time steps.* ◦

Remark 1. *If a system is structurally controllable, then almost all $(\alpha'', A'', B'', T) \in ([\bar{\alpha}], [\bar{A}], [\bar{B}], T)$ are controllable in T time steps, by invoking similar density arguments to those in [4].* ◇

From the above discussion, it readily follows that structural controllability will depend on the system's structure and actuation capabilities being deployed. We consider the following assumption:

A1: All state variables can be directly controlled by dedicated actuators (i.e., there is a one-to-one mapping between an actuator and a state variable). Thus, the input matrix $\mathbb{I}_n^{\mathcal{J}} \in \mathbb{R}^{n \times n}$, where $\mathcal{J} = \{1, \ldots, n\}$ is the set of all state variables, is a diagonal matrix such that any diagonal entry is non-zero (i.e., $\mathbb{I}_n^{\mathcal{J}}(i,i) \neq 0$ where $i = \{1, \ldots, n\}$) if and only if the associated actuator (i.e., u_i) is connected to the associated state variable (i.e., x_i). Hence, the minimum set of state variables that need to be connected to dedicated actuators to ensure structural controllability is denoted by $\mathcal{J}^* \subseteq \mathcal{J}$.

Formally, we seek the solution $\mathcal{J}^*$ to the following problem: given $(\bar{\alpha}, \bar{A})$ and a time horizon T time steps

$$\begin{aligned} \min_{\mathcal{J} \subseteq \{1,\ldots,n\}} \quad & |\mathcal{J}| \\ \text{s.t.} \quad & (\bar{\alpha}, \bar{A}, \mathbb{I}_n^{\mathcal{J}}, T) \\ & \text{is structurally controllable in } T \text{ time steps.} \end{aligned} \tag{$\mathbf{P}_1$}$$

Structural Controllability of Fractional-Order Dynamical Networks

We will start by first providing the graph-theoretical necessary and sufficient conditions to ensure structural controllability in T time steps of fractional-order dynamical networks. With these conditions, we will solve $\mathbf{P}_1$ and provide a characterization of all the minimum combinations of state variables that satisfy these conditions.

Let us start by recalling that the fractional-order dynamical network in (2) can be written as follows [5]:

$$x[k+1] = Ax[k] - \sum_{j=1}^{k+1} D(\alpha, j)x[k+1-j] + Bu[k], \tag{5}$$

where

$$D(\alpha, j) = \begin{bmatrix} \psi(\alpha_1, j) & 0 & \dots & 0 \\ 0 & \psi(\alpha_2, j) & \dots & 0 \\ 0 & \dots & \ddots & 0 \\ 0 & 0 & \dots & \psi(\alpha_n, j) \end{bmatrix}.$$

In fact, it admits a compact representation given by

$$x[k+1] = \sum_{j=0}^{k} A_j x[k-j] + Bu[k], \tag{6}$$

where $A_0 = A - D(\alpha, 1)$ and $A_j = -D(\alpha, j+1)$, for $j \geq 1$. Thus, the fractional-order dynamical network can be re-written in a closed-form as follows:

$$x[k] = G_k x[0] + \sum_{j=0}^{k-1} G_{k-1-j} Bu[j], \tag{7}$$

with

$$G_k = \begin{cases} I_n, & k = 0 \\ \sum\limits_{j=0}^{k-1} A_j G_{k-1-j}, & k \geq 1, \end{cases} \tag{8}$$

Hereafter, the following remark will play a key role.

Remark 2. *The matrix G_k in (8) corresponds to the transition matrix $\Phi(k, 0)$ of the fractional-order dynamical network. In particular, G_k is a combination of the powers of A_0 and diagonal matrices that depend on the fractional-order exponents. For example,*

$$\begin{aligned} G_3 &= \sum_{j=0}^{2} A_j G_{2-j} = A_0 G_2 + A_1 G_1 + A_2 G_0 \\ &= A_0^3 - A_0 D(\alpha, 2) - D(\alpha, 2) A_0 - D(\alpha, 3). \qquad \diamond \end{aligned}$$

To provide necessary and sufficient graph-theoretical conditions, we need to introduce the following terminology. A *directed graph* (digraph) is described by $\mathcal{G} = (\mathcal{V}, \mathcal{E})$, where $\mathcal{V}$ denotes the set of vertices (or nodes) and $\mathcal{E}$ the (directed) edges between the vertices in the graph. A *walk* is any sequence of edges where the last vertex in one edge is the beginning of the next edge. Notice that a walk may include the repetition of vertices. As such, a *path* is a walk where vertices are not repeated. If the beginning and ending vertex of a path is the same, then we obtain a *cycle*. Additionally, a *sub-digraph* $\mathcal{G}_s = (\mathcal{V}', \mathcal{E}')$ is described as any subcollection of vertices $\mathcal{V}' \subset \mathcal{V}$ and the edges between them (i.e., $\mathcal{E}' \subset \mathcal{E}$). If a subgraph has the property that there exists a path between any two pairs of vertices in the subgraph, then it is a *strongly connected digraph*. The maximal strongly connected subgraph forms a *strongly connected component (SCC)*, and any digraph can be uniquely decomposed into SCCs that can be seen as nodes in a directed acyclic digraph. A *source SCC* is an SCC that does not possess incoming edges to its vertices from other SCCs.

Now, we introduce the following notion of *structural equivalence*, which will play a key role in the derivation of our main results.

Definition 3. *(Structural Equivalence) Let $\bar{M}$ and $\bar{N}$ be two $n \times n$ structural matrices. A structural matrix $\bar{M}$ dominates $\bar{N}$ if $\bar{N}_{i,j} = \star$, then $\bar{M}_{i,j} = \star$ for all $i, j \in \{1, \dots, n\}$, which we denote as $\bar{M} \geq \bar{N}$. Also, if $\bar{M} \geq \bar{N}$ and $\bar{N} \geq \bar{M}$, then we say that $\bar{M}$ is structurally equivalent to $\bar{N}$.* $\circ$

We define a Markov network as a linear time-invariant system given as follows

$$x[k+1] = Ax[k],$$

where $A \in \mathbb{R}^{n\times n}$ may or may not possess self-loops on all the nodes.

We notice that for A_j, if $j=0$, then we obtain a system dependent on $A_0 = A - D(\alpha, 1)$; however, the linear time-invariant system is only dependent on A, where A may or may not possess self-loops on all the nodes. When A does not possess self-loops, then we can obtain an advantage in terms of minimal amount of control resources needed for controlling networks possessing fractional-order dynamics. Now, we provide the first main result of our paper.

Theorem 2. *(Structural equivalence of fractional-order dynamical networks to linear time-invariant dynamical networks) The structural fractional-order dynamical network $(\bar{\alpha}, \bar{A})$ described by its transition matrix $\bar{G}_k$ in* (7) *and* (8) *is structurally equivalent to the structural linear time-invariant dynamical network described by system matrix $\bar{A}_0$, where $A_0 = A - D(\alpha, 1)$.* ○

Proof. First recall Remark 2, and notice that if we consider G_k in (8), then we obtain a combination of the powers of A_0 and diagonal matrices that depend on the fractional-order exponents. In fact, some of the powers of A_0 might be multiplied on the left or right by these diagonal matrices, which does not change the structural pattern of the outcome (i.e., DA_0^k or $A_0^k D$ is structurally equivalent to $\bar{A}_0^k$, where D is a diagonal matrix). Therefore, $\bar{G}_k$ structurally equivalent to $\bar{A}_0^k$. For a linear time-invariant system having system matrix A_0, the state transition is described by $x[k] = A_0^k x[0] + \sum_{j=0}^{k-1} A_0^{k-j-1} Bu[j]$. By comparing this state transition relationship with the state transition relationship in (7) and because $\bar{G}_k$ in (8) structurally equivalent to $\bar{A}_0^k$, then the structural fractional-order dynamical network described by $\bar{G}_k$ is structurally equivalent to the structural linear time-invariant network described by system matrix $\bar{A}_0$. □

Next, we show that the structural matrix $\bar{A}_0$ has non-zero diagonal generically.

Theorem 3. *(Generic non-zero diagonal) The structural matrix $\bar{A}_0$ has non-zero diagonal generically.*

Proof. We have by definition that $A_0 = A - D(\alpha, 1)$. A non-zero diagonal entry may appear in A_0 if there exists an $i \in \{1, \ldots, n\}$ such that $\alpha_i = 0$ and if the corresponding diagonal entry of A is zero (i.e., $a_{i,i} = 0$). Another instance occurs if there exists an $i \in \{1, \ldots, n\}$ such that $a_{i,i} \neq 0$, but a given combination of parameters due to $\alpha_i \neq 0$ results in a perfect cancellation of the diagonal entry. These two cases occur with probability zero (whenever they are uniformly sampled on $\mathbb{R}$ or $\mathbb{C}$) by invoking density arguments. Hence, the matrix $\bar{A}_0$ has non-zero diagonal entries generically. □

From Theorems 2 and 3, given the structural characterization, we can associate fractional-order dynamical networks, characterized by (α, A, B), with a *system digraph* $\mathcal{G} \equiv \mathcal{G}(\bar{A}_0, \bar{B}) = (\mathcal{V}, \mathcal{E})$, where $\mathcal{V} = \mathcal{X} \cup \mathcal{U}$ where $\mathcal{X} = \{x_1, \ldots, x_n\}$ and $\mathcal{U} = \{u_1, \ldots, u_n\}$ are the state and input vertices, respectively. Furthermore, we have that $\mathcal{E} = \mathcal{E}_{\mathcal{X},\mathcal{X}} \cup \mathcal{E}_{\mathcal{X},\mathcal{U}}$, where $\mathcal{E}_{\mathcal{X},\mathcal{X}} = \{(x_j, x_i) : \bar{A}_0(i,j) \neq 0\}$ and $\mathcal{E}_{\mathcal{X},\mathcal{U}} = \{(x_j, u_i) : \bar{B}(i,j) \neq 0\}$ are the state and input edges, respectively. Similarly, we can define the *state digraph* $\mathcal{G}(\bar{A}_0) = (\mathcal{X}, \mathcal{E}_{\mathcal{X},\mathcal{X}})$, characterized by (α, A).

Remark 3. *We remark that due to the structural equivalence notion introduced in this paper we observe that the fractional-order exponents play an important role in capturing the memory of the state variables, which is structurally equivalent to nodal dynamics in a linear time-invariant system. Ultimately, by Theorems 2 and 3, considering fractional-order dynamics leads to a system digraph with self-loops almost always.* ◇

Subsequently, by invoking Theorem 2, we provide the graphical conditions that ensure structural controllability of fractional-order dynamical networks.

Theorem 4. *(Structural controllability for fractional-order dynamical networks) Given a structural fractional-order dynamical network $(\bar{\alpha}, \bar{A}, \bar{B}, T = n)$, we say that this network is structurally controllable in $T = n$ time steps if and only if at least one state variable in each of the source SCCs of $\mathcal{G}(\bar{A}_0)$ is connected to an incoming input in the system digraph $\mathcal{G}(\bar{A}_0, \bar{B})$.* ○

Proof. From Theorems 2 and 3, it follows that we only need to guarantee that the linear time-invariant network described by $(\bar{A}_0, \bar{B})$ is structurally controllable. Therefore, to attain structural controllability of $(\bar{A}_0, \bar{B})$, we need to guarantee two conditions on $\mathcal{G}(\bar{A}_0, \bar{B})$ [6]: (i) all state variables belong to a disjoint union of cycles, and (ii) $\mathcal{G}(\bar{A}_0, \bar{B})$ has at least one state variable in each of the source SCCs of $\mathcal{G}(\bar{A}_0)$ that is connected to an incoming input. Notice that the first condition is fulfilled since all the states have self-loops generically – see Remark 3. Subsequently, it suffices to guarantee structural controllability of the fractional-order dynamical network if and only if $\mathcal{G}(\bar{A}_0, \bar{B})$ has at least one state variable in each of the source SCCs of $\mathcal{G}(\bar{A}_0)$ that is connected to an incoming input. □

Minimal Dedicated Actuation to Ensure Structural Controllability of Fractional-Order Dynamical Networks

With the result in Theorem 4, we readily obtain the following corollary required for ensuring the feasibility of $\mathbf{P}_1$.

Corollary 1. *A fractional-order dynamical network* $(\bar{\alpha}, \bar{A}, \mathbb{I}_n^{\mathcal{J}}, T = n)$ *is structurally controllable if and only if* $\mathcal{J}$ *contains the index of at least one state variable in each of the source SCCs in* $\mathcal{G}(\bar{A}_0)$. ∘

Proof. The result follows from invoking Theorem 4. Therefore, by guaranteeing that at least one state per source SCC is actuated, we guarantee that $\mathcal{G}(\bar{A}_0, \mathbb{I}_n^{\mathcal{J}})$ is accessible and hence, structurally controllable. □

Consequently, we obtain the solution to $\mathbf{P}_1$.

Theorem 5. *(Solution to* $\mathbf{P}_1$*) Consider a fractional-order dynamical networks* $(\bar{\alpha}, \bar{A}, \mathbb{I}_n^{\mathcal{J}}, T = n)$. *The solution to* $\mathbf{P}_1$ *is as follows:*

$$\mathcal{J}^* = \{i_1, \ldots, i_l\},$$

where $\{i_1, \ldots, i_l\}$ *denotes the set of indices corresponding to the* l *states* $x_{i_1}, \ldots, x_{i_l}$ *that each belong to a different source SCC in* $\mathcal{G}(\bar{A}_0)$. ∘

Proof. First, notice that Corollary 1 establishes the feasibility of the solution to $\mathbf{P}_1$. Therefore, to achieve the minimum feasible set, we select one state variable from each of the different source SCCs in $\mathcal{G}(\bar{A}_0)$ to be actuated. The minimal number of variables is equal to the number of source SCCs, and hence, the result follows. □

Theorem 6. *Any network modeled as a fractional-order system as in* (1) *requires less than or equal to the number of driven nodes than that of the same network possessing linear time-invariant dynamics.*

Proof. Based on the results in Theorem 5 and the results in [7], linear time-invariant networks have one more additional condition to verify structural controllability than the sole condition required for fractional-order networks. Therefore, a network possessing linear time-invariant dynamics must have the same or more total number of driven nodes than the equivalent topological network possessing fractional-order dynamics. □

Finally, we provide the computational-time complexity for solving $\mathbf{P}_1$.

Theorem 7. *The computational-time complexity of the solution to* $\mathbf{P}_1$ *is given as* $\mathcal{O}(n^2)$.

Proof. Based on Theorem 5, the solution to $\mathbf{P}_1$ depends on finding the source strongly connected components. Tarjan's algorithm finds all the strongly connected components in a directed network with a computational-time complexity of $\mathcal{O}(|\mathcal{V}| + |\mathcal{E}|)$ [8], where $\mathcal{V}$ is the number of vertices and $\mathcal{E}$ is the number of edges in the network. Hence, by performing another pass of depth-first search, which also has a computational-time complexity of $\mathcal{O}(|\mathcal{V}| + |\mathcal{E}|)$, then the strongly connected components that do not have an incoming edge can be identified, which are the source strongly connected components. We notice that $\mathcal{E} \subseteq \mathcal{V} \times \mathcal{V}$. Hence, $\mathcal{O}(|\mathcal{V}| + |\mathcal{E}|) = \mathcal{O}(|\mathcal{V}|^2) = \mathcal{O}(n^2)$, and the result follows. □

Minimal Dedicated Actuation to Ensure Structural Controllability of Fractional-Order Dynamical Networks in a Given Number of Time Steps $T < n$

Next, we will we provide the solution to find the minimum combination of state variables that ensure the structural controllability of fractional-order dynamical networks with a given number of time steps $T < n$, which is written as follows

$$\begin{aligned} \min_{\mathcal{J} \subseteq \{1,\ldots,n\}} \quad & |\mathcal{J}| \\ \text{s.t.} \quad & (\bar{\alpha}, \bar{A}, \bar{\mathbb{I}}_n^{\mathcal{J}}, T < n) \text{ is structurally controllable.} \end{aligned} \tag{$\mathbf{P}_2$}$$

In the next result, we provide a solution to $\mathbf{P}_2$.

Theorem 8. *(Structural controllability for fractional-order dynamical networks with a given horizon $T < n$) A fractional-order dynamical network $(\bar{\alpha}, \bar{A}, \bar{B}, T < n)$, is structurally controllable for a given horizon $T < n$ if and only if the following two conditions are satisfied:*

1. *there is at least one state variable in each source SCC in $\mathcal{G}(\bar{A}_0)$ connected to an input, and*
2. *the length of the longest shortest path from the starting node of any source SCC in $\mathcal{G}(\bar{A}_0, \bar{B})$ is less than or equal to T.*

$\circ$

Proof. The first condition follows directly from Theorem 4. The second condition ensures that the system is controllable in $T < n$ time steps since the network can only communicate information as fast as the longest shortest path from the input to the last node in the network. □

While Theorem 8 does provide an exact solution to $\mathbf{P}_2$, this solution is NP-hard. We prove this claim in the next result.

Theorem 9. *Problem $\mathbf{P}_2$ is NP-hard.*

Proof. We need to show that there exists a polynomial reduction from a problem known to be NP-hard to our problem. The known NP-hard problem that we consider is the graph partitioning problem [9], which aims to determine the minimum decomposition of $\mathcal{G} = (\mathcal{V}, \mathcal{E})$ into p connected directed subgraphs $\mathcal{G}_i = (\mathcal{V}_i, \mathcal{E}_i)$, with $i \in \{1, \ldots, p\}$ such that $|\mathcal{V}_i| \leq T$, $\mathcal{V}_i \cap \mathcal{V}_j = \emptyset$ for $i \neq j$ and $\bigcup_{i=1}^{p} \mathcal{V}_i = \mathcal{V}$. If we partition the network $\mathcal{G}(\bar{A}_0)$ into p subgraphs such that each subgraph has $|\mathcal{V}_i| \leq T$, then we can ensure that the longest shortest path from the starting node of any source SCC in each subgraph is less than or equal to T because each subgraph has at most T nodes, which satisfies condition 2 in Theorem 8. Furthermore, the source SCCs can be found in polynomial time [10], which satisfies condition 1 of Theorem 8. Together, this method provides a solution to $\mathbf{P}_2$. Hence, our problem is at least as difficult as the graph partitioning problem, which is known to be NP-hard, so $\mathbf{P}_2$ is NP-hard. □

Since $\mathbf{P}_2$ cannot be solved exactly, we propose an approximated solution to $\mathbf{P}_2$, which is employed in our simulations and shown in Algorithm 1. Briefly, Algorithm 1 takes a fractional-order dynamical network and a given number of time steps $T < n$ and finds the minimum set of state variables $\mathcal{J}$ to ensure structural controllability. First, the algorithm computes the digraph from the fractional-order dynamical network. Next, the software package METIS [9] is used to partition the graph into $\lceil \frac{n}{T} \rceil$ subgraphs of roughly equal size T. Finally, all of the source SCCs are found in each subgraph, and a single node from each source SCC is added to the set $\mathcal{J}$.

Next, we provide an lower-bound on the optimal solution to $\mathbf{P}_2$.

Algorithm 1: Find the minimum set of state variables $\mathcal{J}$ to ensure structural controllability of fractional-order dynamical networks for a given time horizon $T < n$

Input: Fractional-Order Dynamical Network $(\bar{\alpha}, \bar{A}, T)$ and network size n
Output: The set of state variables denoted by $\mathcal{J} \subseteq \{1, \ldots, n\}$
Initialization: Compute $\mathcal{G}(\bar{A}_0)$ from the fractional-order dynamical network $(\bar{\alpha}, \bar{A}, T)$ and network size n
Step 1: Using METIS [9], partition the digraph $\mathcal{G}(\bar{A}_0)$ into $\lceil \frac{n}{T} \rceil$ partitions denoted by $\mathcal{G}_i = (\mathcal{V}_i, \mathcal{E}_i)$, where each partition is roughly equal sized, i.e. $|V_i| \leq T$.
Step 2: Find all the source SCCs $\mathcal{S}_{i,j}$ in each partition $\mathcal{G}_i$, where j is the index of all the source SCCs in subgraph $\mathcal{G}_i$
Step 3: Add one state from each source SCC $\mathcal{S}_{i,j}$ to the set $\mathcal{J}$.

Theorem 10. *The minimum number of driven nodes d required to solve $\mathbf{P}_2$ for a given time horizon T is given by the following inequality:*

$$d \geq \left\lceil \frac{n}{T} \right\rceil. \tag{9}$$

Proof. When partitioning the graph into subgraphs, we ensure each subgraph has $|\mathcal{V}_i| \leq T$. Therefore, there are a maximum of $\lceil \frac{n}{T} \rceil$ subgraphs. Each subgraph can have a minimum of only one source SCC, so the lower-bound on the number of driven nodes is equal to the number of subgraphs, i.e., $\lceil \frac{n}{T} \rceil$. □

Finally, we present the computational-time complexity of Algorithm 1.

Theorem 11. *The computational-time complexity of Algorithm 1 is given as $\mathcal{O}(n^2 \log(n))$.*

Proof. The complexity of this sequential algorithm is determined by the step that has the maximum computational-time complexity. The initialization step has a complexity of $\mathcal{O}(n^2)$ since we construct the network from its adjacency matrix $\bar{A}_0$. Step 1 has a computational time-complexity of $\mathcal{O}(n^2 \log(n))$ [11]. Step 2 has a computational-time complexity of $\mathcal{O}(n^2)$ [8]. Step 3 has a computational-time complexity of $\mathcal{O}(n)$ since we select a single node out of all the nodes in a source SCC, which could be possibly n nodes. Hence, Step 1 has the largest computational-time complexity, so this dictates the overall complexity of the algorithm, and the result follows. □

2 Extra Experiments

We investigate the relationship between the average degree and the average difference in the required number of driven nodes for the three random networks. The results are shown in Figure 1. With the exception of the Watt-Strogatz networks, which have the same degree for each of the generated networks, the average difference in the required number of driven nodes stays relatively similar as the average degree of the network increases.

We examine the rat brain network since this gave the highest difference in required number of driven nodes. In particular, we examine the degree distribution and clustering coefficient distribution for the rat brain network to gain insight as to why this network gives such a significant improvement in the required number of driven nodes when considering the fractional-order dynamical network model – see Figure 2 (b) and (c). We notice that the rat brain network has wide range of degrees and a fairly high clustering coefficient. We conjecture that these properties play a role in achieving a high difference in driven nodes.

Using the progressive ChungLu method developed in [12], we generate 100 networks that are on average similar in degree distribution to the rat brain network. From the results in Figure 2 (a), we see that the mean and standard deviation of the difference in driven nodes for the generated networks drastically differ the results for the original rat brain network. As a way to understand why we see this drastic difference in results, we performed the Spearman Rank Test (Chapter 8.5, [13]), which tests whether any two real-valued vectors of equal length are independent. In particular, the null hypothesis states that the two vectors are

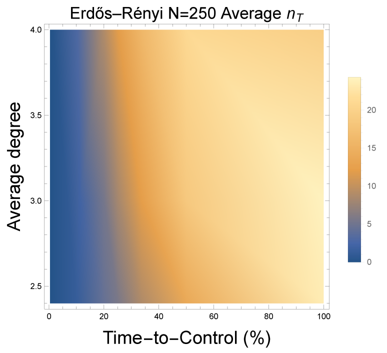

(a)

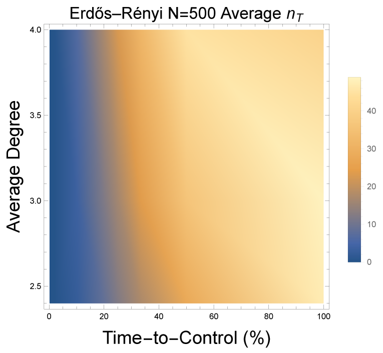

(b)

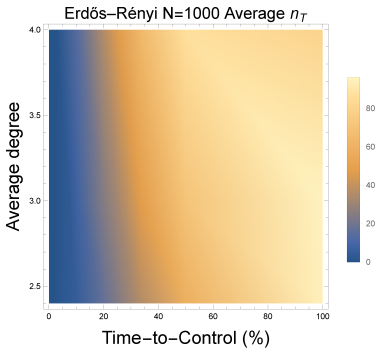

(c)

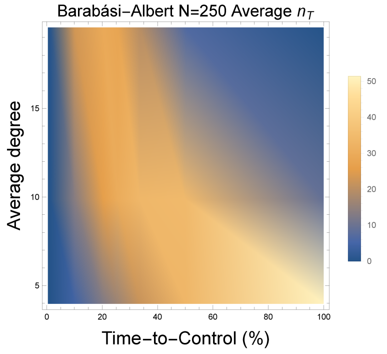

(d)

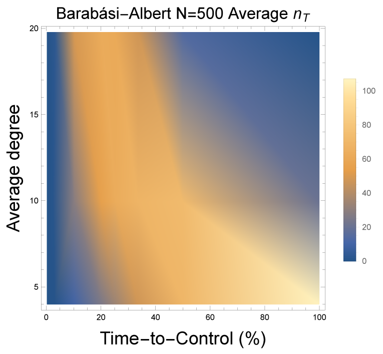

(e)

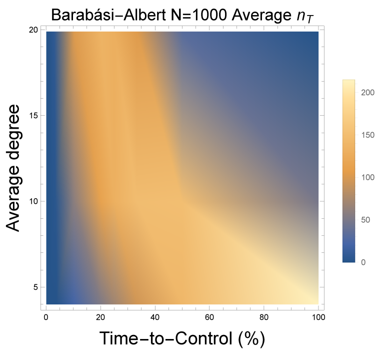

(f)

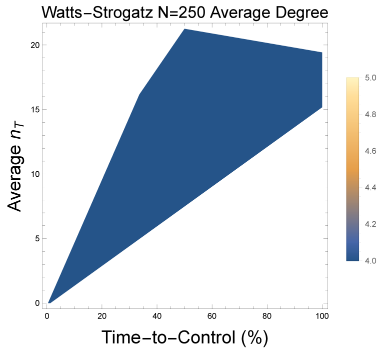

(g)

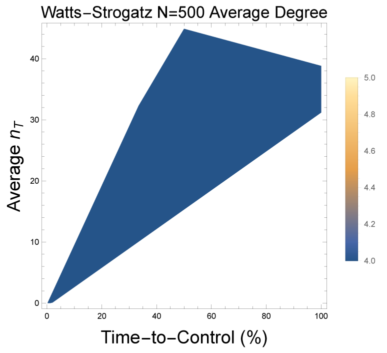

(h)

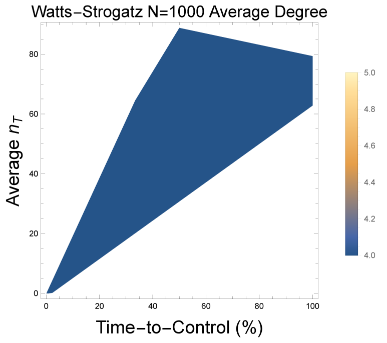

(i)

Figure 1: For networks of sizes 250, 500, and 1000 nodes, respectively, Figures (a), (b), and (c) show the average difference in the required number of driven nodes (n_T) for networks of varying average degree distributions versus the time-to-control (%) for 100 realizations of Erdős–Rényi networks. For networks of sizes 250, 500, and 1000 nodes, respectively, Figures (d), (e), and (f) show the average difference in the required number of driven nodes (n_T) for networks of varying average degree distributions versus the time-to-control (%) for 100 realizations of Barabási–Albert networks. For networks of sizes 250, 500, and 1000 nodes, respectively, Figures (g), (h), and (i) show the average degree of networks versus the required number of driven nodes (n_T) across the time-to-control (%) for 100 realizations of Watts-Strogatz networks. In the case of the Watts-Strogatz networks, we notice that the average degree is the same for all of the networks.

indeed independent. Hence, if the p-value is large, then the null hypothesis is accepted, whereas if the p-value is small, then the null hypothesis is rejected. For each of the 100 generated networks, we compare the distribution of the in-degree, out-degree, and total degree for the generated network with those for the rat brain network.

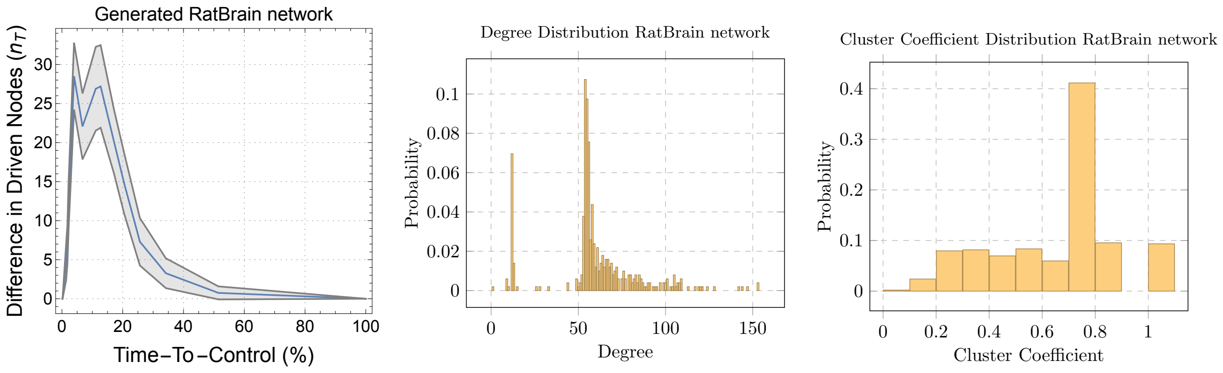

(a) (b) (c)

Figure 2: (a) shows the mean and standard deviation of the difference in driven nodes versus time-to-control for 100 networks generated from the rat brain network following the progressive Chung Lu method [12]. (b) shows the degree distribution for the original rat brain network. (c) shows the clustering coefficient for the original rat brain network.

First, we provide the results of the Spearman Rank Test when considering the in-degree distribution. With 99% confidence, only 55% of the generated networks have vertex in-degree distributions that are independent of the vertex in-degree distribution for the rat brain network. With 95% confidence, 80% of the generated networks have vertex in-degree distributions that are independent of the vertex in-degree distribution for the rat brain network. With 90% confidence, 87% of the generated networks have vertex in-degree distributions that are independent of the vertex in-degree distribution for the rat brain network. Therefore, we can say with high confidence that most of the generated networks have in-degree distributions that are independent from the in-degree distribution of the rat brain network. This may provide an explanation as to why the difference in the number of driven nodes needed for the generated networks differs drastically from difference in the required number of driven nodes for the rat brain network.

Next, we provide the results of the Spearman Rank Test when considering the out-degree distribution. With 99% confidence, only 26% of the generated networks have vertex out-degree distributions that are independent of the vertex out-degree distribution for the rat brain network. With 95% confidence, 52% of the generated networks have vertex out-degree distributions that are independent of the vertex out-degree distribution for the rat brain network. With 90% confidence, 63% of the generated networks have vertex out-degree distributions that are independent of the vertex out-degree distribution for the rat brain network. Surprisingly, we can say with high confidence that very few of the generated networks have out-degree distributions that are independent from the out-degree distribution of the rat brain network.

Finally, we provide the results of the Spearman Rank Test when considering the total degree distribution. With 99% confidence, 70% of the generated networks have total degree distributions that are independent of the total degree distribution for the rat brain network. With 95% confidence, 94% of the generated networks have total degree distributions that are independent of the total degree distribution for the rat brain network. With 90% confidence, 94% of the generated networks have total degree distributions that are independent of the total degree distribution for the rat brain network. Therefore, we can say with high confidence that more than 70% of the generated networks have total degree distributions that are independent from the total degree distribution of the rat brain network. This provides evidence to support that the difference in the number of driven nodes needed for the generated networks would differ drastically from the difference in the required number of driven nodes for the rat brain network.

References

[1] I. Podlubny, "Chapter 2 - fractional derivatives and integrals," in *Fractional Differential Equations: An Introduction to Fractional Derivatives, Fractional Differential Equations, to Methods of their Solution*

and some of their Applications, ser. Mathematics in Science and Engineering. Elsevier, 1999, vol. 198, pp. 41–119. [Online]. Available: https://www.sciencedirect.com/science/article/pii/S0076539299800216

[2] A. Dzielinski and D. Sierociuk, "Adaptive Feedback Control of Fractional Order Discrete State-Space Systems," in *Proceedings of the International Conference on Computational Intelligence for Modelling, Control and Automation*, vol. 1, Nov 2005, pp. 804–809.

[3] S. Guermah, S. Djennoune, and M. Bettayeb, "Controllability and observability of linear discrete-time fractional-order systems," *International Journal of Applied Mathematics and Computer Science*, vol. 18, no. 2, pp. 213–222, 2008.

[4] S. Pequito, P. Bogdan, and G. J. Pappas, "Minimum number of probes for brain dynamics observability," in *Proceedings of the 54th IEEE Conference on Decision and Control.* IEEE, 2015, pp. 306–311.

[5] E. Reed, S. Chatterjee, G. Ramos, P. Bogdan, and S. Pequito, "Fractional cyber-neural systems—a brief survey," *Annual Reviews in Control*, 2022.

[6] G. Ramos, A. P. Aguiar, and S. Pequito, "An overview of structural systems theory," *Automatica*, vol. 140, p. 110229, 2022.

[7] S. Pequito, S. Kar, and A. P. Aguiar, "A framework for structural input/output and control configuration selection in large-scale systems," *IEEE Transactions on Automatic Control*, vol. 61, no. 2, pp. 303–318, 2015.

[8] R. Tarjan, "Depth-first search and linear graph algorithms," *SIAM Journal on Computing*, vol. 1, no. 2, pp. 146–160, 1972.

[9] G. Karypis and V. Kumar, "A fast and high quality multilevel scheme for partitioning irregular graphs," *SIAM Journal on Scientific Computing*, vol. 20, no. 1, pp. 359–392, 1998.

[10] E. A. Reed, G. Ramos, P. Bogdan, and S. Pequito, "A scalable distributed dynamical systems approach to compute the strongly connected components and diameter of networks," *IEEE Transactions on Automatic Control*, 2022.

[11] B. W. Kernighan and S. Lin, "An efficient heuristic procedure for partitioning graphs," *The Bell System Technical Journal*, vol. 49, no. 2, pp. 291–307, 1970.

[12] G. Ramos and S. Pequito, "Generating complex networks with time-to-control communities," *PloS One*, vol. 15, no. 8, p. e0236753, 2020.

[13] M. Hollander, D. A. Wolfe, and E. Chicken, *Nonparametric statistical methods.* John Wiley & Sons, 2013.
